# Plasma and Myocardial miRNomes Similarities and Differences during Cardiac Remodelling and Reverse Remodelling in a Murine Model of Heart Failure with Preserved Ejection Fraction

**DOI:** 10.3390/biom14080892

**Published:** 2024-07-24

**Authors:** Sara-Ève Thibodeau, Emylie-Ann Labbé, Élisabeth Walsh-Wilkinson, Audrey Morin-Grandmont, Marie Arsenault, Jacques Couet

**Affiliations:** Groupe de Recherche sur les Valvulopathies, Institut Universitaire de Cardiologie et de Pneumologie de Québec, Université Laval, Quebec City, QC G1V 4G5, Canada; sara-eve.thibodeau@criucpq.ulaval.ca (S.-È.T.); emylie-ann.labbe@criucpq.ulaval.ca (E.-A.L.); elisabeth.walsh-wilkinson@criucpq.ulaval.ca (É.W.-W.); audrey.morin-grandmont@criucpq.ulaval.ca (A.M.-G.); marie.arsenault@criucpq.ulaval.ca (M.A.)

**Keywords:** mouse, heart failure, myocardial recovery, reverse remodelling, cardiac hypertrophy, microRNA, preclinical model

## Abstract

Heart failure with preserved ejection fraction (HFpEF) is a heterogeneous syndrome characterised by multiple risk factors touching various organs outside the heart. Using a murine HFpEF model, we studied cardiac reverse remodelling (RR) after stopping the causing metabolic-hypertensive stress (MHS; Angiotensin II [AngII] and a high-fat diet [HFD]) after 28 days and introducing voluntary exercise (VE) for four more weeks. We measured the effects of MHS and RR on the plasma and myocardial microRNA (miR) profile (miRNome) to characterise better cardiac and non-cardiac responses to HFpEF-inducing risk factors and their reversibility. AngII alone, the HFD or the MHS caused cardiac hypertrophy (CH), left ventricular (LV) concentric remodelling and left atrial enlargement in females. Only AngII and the MHS, but not HFD, did in males. After RR, CH, LV concentric remodelling and atrial enlargement were normalised. Among the 25 most abundant circulating miRs, 10 were modulated by MHS. Plasma miRNomes from AngII, HFD or MHS mice shared 31 common significantly modulated miRs (24 upregulated and 7 downregulated), suggesting that the response of organs producing the bulk of those circulating miRs was similar even for seemingly different stress. In the LV, 19 out of 25 most expressed miRs were modulated. RR restored normality for the plasma miRNome but not for the LV miRNome, which remained mostly unchanged. Our results suggest that abnormalities persist in the myocardium of the HFpEF mice and that the normalisation of circulatory markers may be falsely reassuring after recovery.

## 1. Introduction

Heart failure (HF) is divided into three basic categories based on left ventricular (LV) ejection fraction (EF): HF with preserved ejection fraction (HFpEF; EF ≥ 50%), HF with reduced EF (HFrEF; EF < 40%), and HF with midrange EF (HFmEF, EF ≥ 40 and <50%) [1]. Patients with HFpEF are often older, more of them female, hypertensive and obese [2]. Patients with HFpEF have higher rates of morbidities, mortality, and rehospitalisations than those with HFrEF and have poorer quality of life [3].

HFpEF is a multifactorial disease displaying various phenotypes. Age, hypertension, and female sex are classical risk factors contributing to its development. Atrial fibrillation is also present in a significant portion of patients suffering from HFpEF. A cardiometabolic phenotype, including obesity and type 2 diabetes mellitus, has become more prevalent in recent years [4,5].

The development of efficient medical therapies for HFpEF has lagged behind the one made for HFrEF, where many drugs have been proven helpful in reducing the burden of symptoms and improving survival [6]. The recent EMPEROR-Preserved clinical trial has yielded encouraging results, showing for the first time that a medication (SGLT2 inhibitors) could benefit significant clinical endpoints in HFpEF [7]. This has come after years of unsuccessful trials with many classes of drugs that sometimes had shown clear benefits in HFrEF.

Aerobic exercise training has emerged as one of the most effective means of improving outcomes in patients with HFpEF. Therapeutic strategies for managing this population should combine a pharmacological intervention with one targeting lifestyle changes. Multiple randomised clinical trials of supervised exercise training in selected patients with chronic and stable HFpEF showed that exercise could provide clinically relevant improvements in exercise capacity and quality of life. Lifestyle changes constantly show improvement in studies conducted in HFpEF patients [8].

The search for specific biomarkers has been an ever-expanding research effort to assess the efficacy of these interventions [9]. Among potential circulatory biomarkers, microRNAs (miRs) have emerged as exciting targets and possible therapeutic agents for HF [10]. MiRs are small (22 nucleotides) non-coding RNA able to repress messenger RNA translation or cause its degradation [11]. The underlying dysregulation of the cardiac transcriptome or the miRNome in myocardial remodelling, reverse remodelling (RR), or HF has been studied in the past [12,13,14,15,16,17]. Several studies have described the expression of different sets of miRs in the myocardium and circulation of human HF patients [18,19,20,21]. Comparing those two miRNomes after myocardial remodelling and RR has received less attention, especially in HFpEF.

The availability of suitable HFpEF animal models reflecting different phenotypes has progressed significantly in recent years. In the mouse, HFpEF models triggered by a single factor (hypertension, obesity/diabetes or ageing) have been replaced by more complex models combining more than one factor [22].

Here, we use a “two-hit” murine HFpEF (Angiotensin II (AngII) + High-Fat Diet (HFD); MHS) to study whether stopping AngII, normalising the diet, and introducing voluntary exercise (VE) could help reverse cardiac damage and improve exercise intolerance. In addition, we aimed to better describe the plasma and myocardial miRNomes’ response to stress and to characterise its eventual normalisation afterwards [23]. Our main aim was to potentially identify miR markers of myocardial reverse remodelling.

MHS for 28 days in 8-week-old male and female mice induces an HFpEF phenotype characterised by cardiac hypertrophy (CH), concentric LV remodelling, left atrial enlargement, and reduced exercise capacity. After stopping MHS, four additional weeks normalised CH, concentric LV remodelling, and increased exercise capacity. We observed extensive changes in the plasma and myocardial miRNomes after MHS. Only the plasma miRNome was normalised after RR, whereas the myocardial miRNome remained abnormal.

## 2. Materials and Methods

### 2.1. Animals

C57BL6/J male and female 7-week-old mice were purchased from Jackson Laboratory (Bar Harbor, ME, USA). Mice were housed on a 12 h light–12 h dark cycle with free access to chow and water. The protocol was approved by the Université Laval’s animal protection committee and followed the recommendations of the Canadian Council on Laboratory Animal Care (#2020-603 and #2023-1250).

### 2.2. Experimental Design

Mice were randomly distributed in the various experimental groups. We used a two-hit HFpEF model we recently described [23]. A. Forty-eight two-month-old mice were divided into four groups (six males (M) and six females (F). Half of the animals were implanted with an osmotic minipump providing a continuous infusion of angiotensin II (AngII; 1.5 mg/kg/day) (Sigma, Tokyo, Japan) for 28 days and fed with a high-fat diet (HFD: 60% calories from fat, 20% from protein and 20% from carbohydrates; Research Diets Cat. #D12492). The standard diet had a low-fat content of 10% calories (D12450J). These mice were followed by echocardiography (echo) at baseline (8-week-old) and the day before euthanasia. B. A second batch of mice (24 animals) received either AngII or fed the HFD. C. A third batch of mice received AngII + HFD (MHS) for four weeks. Then, on day 28, osmotic minipumps were removed, and the diet of HFD-fed mice was changed to the standard low-fat diet (RR). The same day, a flying saucer-type exercise device was introduced in their cage (Innowheel™, Innovive, Billerica, MA, USA). Twenty-eight days later, the protocol was stopped. Mice had an echo exam on day 55 and were euthanatised the day after. The heart was weighted and harvested. The animal’s behaviour was monitored daily by experienced technicians for health and behaviour during the protocol. The animals were weighed weekly. No mouse displayed signs associated with poor prognosis of quality of life or specific signs of severe suffering or distress. Among those signs, significant loss or gain of weight, grooming and changes in behaviour were recorded.

### 2.3. Echocardiography

Echocardiography, osmotic mini-pump surgery, and euthanasia were performed under isoflurane anaesthesia as described previously [23,24,25]. The same investigator, blinded for mouse identification, acquired Echo images on a Vevo 3100 imaging system (VisualSonics, FujiFilm, Toronto, ON, Canada). Transthoracic echocardiography was performed using a 40 MHz image transducer (MX550S; VisualSonics, FujiFilm).

### 2.4. Exercise Capacity

Mice were acclimated to treadmill running for three days. Seven days before euthanasia, an exhaustion test was performed as previously described [23]. Running time and distance were recorded.

### 2.5. RNA Isolation

Total RNA from LV tissue was extracted using TRI Reagent (Sigma, Mississauga, ON, Canada), as described previously [23]. Plasma (200 µL) total RNA, including miRNA, was extracted using the Qiagen miRNeasy Serum/Plasma Advanced Kit following suppliers’ instructions (Qiagen, Inc., Toronto, ON, Canada).

### 2.6. RNA Preparation for Sequencing

Immediately after total RNA extraction, DNAse treatment of LV sample, long RNA (>200 bp, mRNA), and small RNA (<200 bp) were separated using different ethanol percentage solutions and Qiagen mini-Kit columns. When necessary, samples were cleaned using the Qiagen RNeasy MiniElute Cleanup Kit to remove genomic DNA traces. RNA integrity and quality (RIN averaging 8.8 (8.1–9.1)) were acquired using Agilent Technologies 2100 Bioanalyzer, namely using an RNA-specific chip (Agilent Eukaryote Total RNA Nano 1000, Santa Clara, CA, USA). To measure complementary DNA (cDNA) library concentrations, the Kapa Universal Library Quantitation kit was used (Roche Sequencing, Mississauga, ON, Canada).

### 2.7. Bulk microRNA-Sequencing

RNA samples were processed with the Qiagen miRNA Library kit following the manufacturer’s recommendations. Each sample was constituted of RNA from one mouse, either male or female. Sequencing libraries were analysed using an Agilent Bioanalyzer to verify their size. An aliquot of the library was run on a 6% PAGE gel, and a band at 175 bp corresponding to the expected size of the library was excised and purified. Indexed libraries were pooled and sequenced on the Illumina NextSeq 2000 (single-end 75 bp) according to the manufacturer’s protocols. An average number of 6.8 million reads per sample was obtained. Micro RNA-Seq Alignment was processed on the Qiagen RNA portal for sequence alignment using the Mus musculus miRBase version 22 (GRCm38.101) reference genome. Around 80% of UMI reads were annotated with the miRBase for left ventricle samples and 15% for plasma samples. The differentially regulated microRNAs were identified with a false discovery rate (FDR) ≤ 0.05, a Log2 fold change (Log2FC), |Log2 FC| ≥ 1 and a normalised count exceeding 10. Volcano plots and heat maps were then generated using this Qiagen platform.

### 2.8. MicroRNA Analyses

The miRNet (www.mirnet.ca) tools were used to determine potential miRNA–target interactions and build network-based analysis (Accessed on 3 August 2023). For the modulated plasma miRs, we used the TargetScan database to determine possible targets. For the myocardial miRs, we created a functional network by pairing upregulated miRs with differentially regulated LV genes after MHS from a previous study [23]. The same was conducted for downregulated miRs [26,27]. Cytoscape software was used to build the illustration [28].

### 2.9. Statistical Analysis

All data are expressed as mean ± standard error of the mean (SEM). Intergroup comparisons were conducted using students’ *t*-tests using GraphPad Prism 10 (GraphPad Software Inc., La Jolla, CA, USA). Comparisons of more than two groups were analysed using one-way or two-way ANOVA and Holm–Sidak post-test. *p* < 0.05 was considered statistically significant.

## 3. Results

### 3.1. Angiotensin II Alone or Combined with a High-Fat Diet Induces Cardiac Hypertrophy in Male and Female Mice

**Figure 1:**
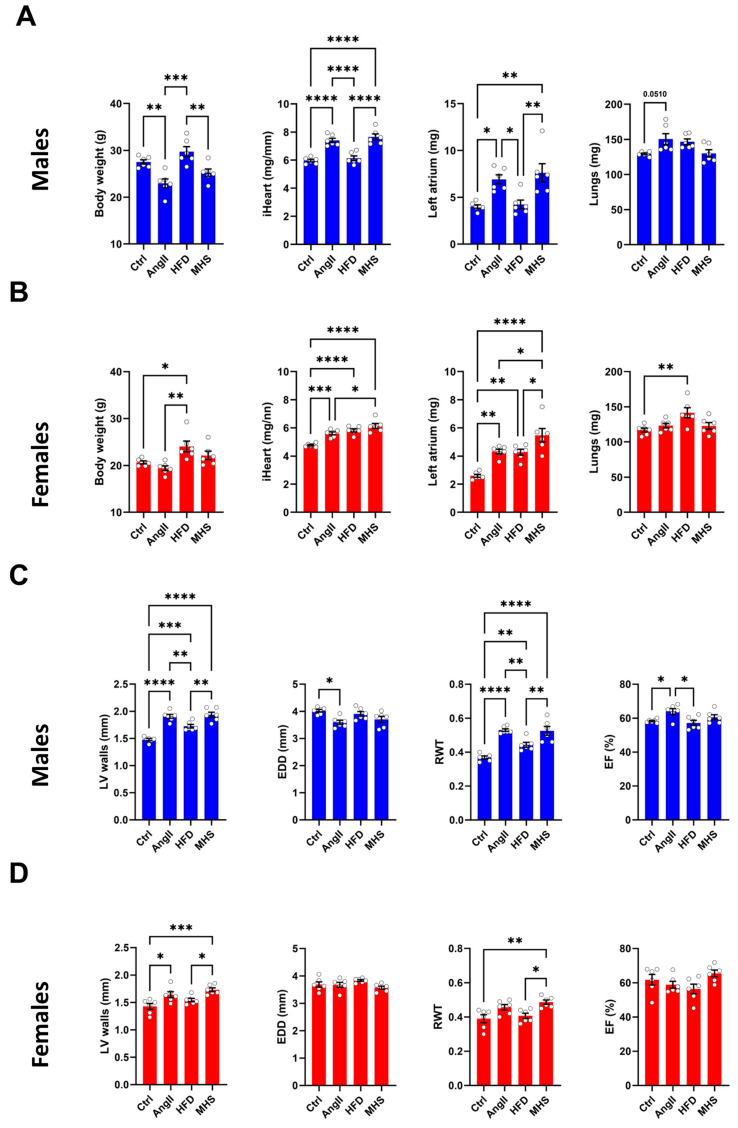
AngII alone or combined (MHS) with HFD induces cardiac hypertrophy and left atrial enlargement in male and female mice. (**A**) Males. Data at euthanasia (n = 6/group). AngII and MHS increased heart and left atrial weight but not lung weight. (**B**) Females. HFD causes obesity, cardiac hypertrophy, left atrial enlargement and lung congestion. AngII and MHS increased heart and left atrial weight. (**C**) Males. Echo data. AngII and/or HFD increased LV wall thickness and relative wall thickness (RWT) without reducing ejection fraction (EF). (**D**) Females. MHS had the strongest effects on LV wall thickening and RWT. Data are represented as mean +SEM. One-way ANOVA followed by Holm–Sidak post-test. * *p* < 0.05, ** *p* < 0.01, *** *p* < 0.001 and **** *p* < 0.0001 between indicated groups.

Male and female mice (n = 6/group) were administered AngII alone or in combination with an HFD for 28 days. In males, AngII reduced body weight, whereas in females, HFD did the opposite (Figure 1A,B). When combined (MHS), body weight remained unchanged.

After 28 days, the MHS significantly increased both males’ and females’ heart and left atrial weights. HFD only caused CH and left atrial enlargement in females (Figure 1B). Lung congestion was evaluated by weighing lungs at the time of euthanasia. Only in females fed with the HFD did we observe an increase in lung weight.

An echo exam was performed the day before euthanasia. Combined interventricular septal wall and posterior wall thickness (LV walls) were increased by AngII, HFD and MHS in males and only by AngII and MHS in females (Figure 1C,D). End-diastolic LV diameter (EDD) remained unchanged in males but was reduced by MHS in females. This resulted in an increased relative LV wall thickness ratio (RWT) in both AngII or MHS males and females. Ejection fraction (EF) was unchanged for all groups. Additional echo data are included in the supplementary Tables S1 and S2.

### 3.2. Plasma miRNomes of Mice Receiving Either AngII or HFD or a Combination of Both (MHS) Show Many Common Modulated microRNAs

**Figure 2:**
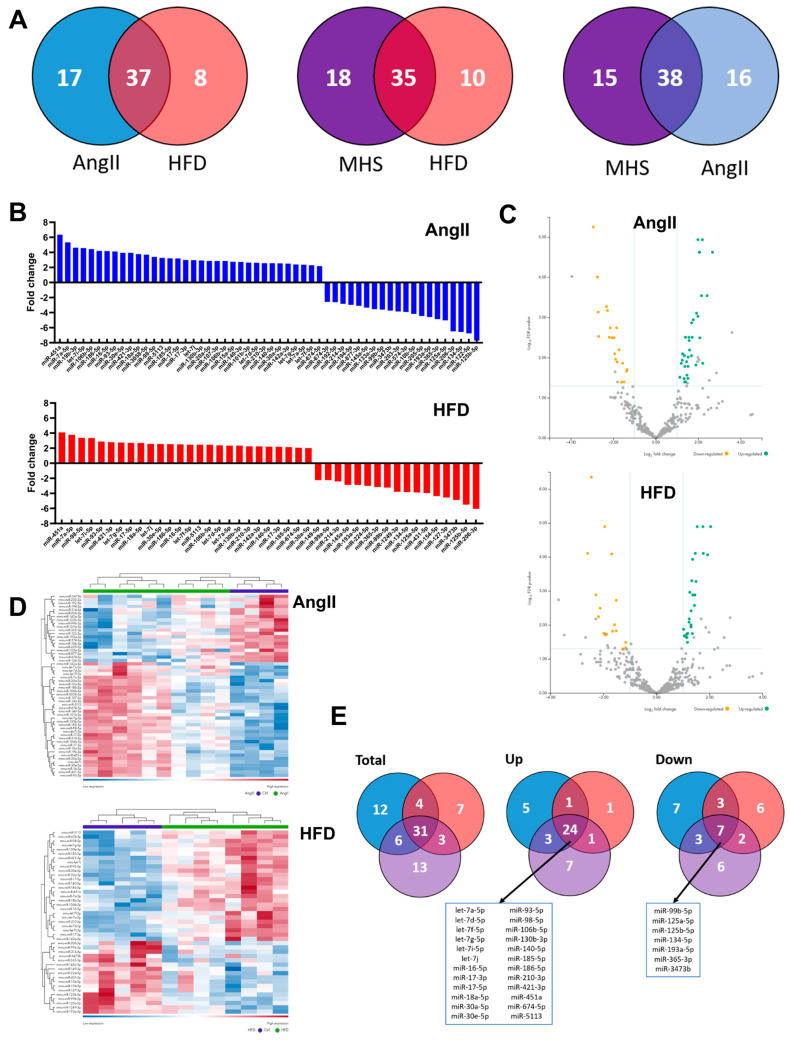
Alterations of the plasma miRNomes after four weeks of AngII, HFD or MHS. (**A**) Venn diagrams illustrating the number of modulated miRs between the control group and the indicated stress group. (AngII: blue, HFD: red or MHS: purple). Many modulated miRs between each stress group were common. (**B**) Upregulated and downregulated circulating miR after four weeks of AngII (blue) or HFD (red). Graphs represent fold change (log_2_) of all significantly modulated miRs (FDR < 0.05) after four weeks of the indicated stress. (**C**) Volcano plots of differently expressed miRs after AngII (up) or HFD (bottom). Green dots illustrate upregulated miRs, and yellow dots downregulated ones. Grey dots had a fold change below ±2 or a false discovery rate (FDR) over 0.5. (**D**) Heat maps of modulated miRs after AngII (up) and HFD (bottom) stress compared to control mice. Purple samples are controls, and green ones are AngII or HFD. Colours range from blue (low expression) to red (high expression). (**E**) Venn diagrams of modulated miRs in AngII (blue), HFD (red) and MHS (purple) compared to control animals. Thirty-one miRs are shared between the three stresses. Twenty-four are upregulated, and seven are downregulated. These miRs are listed below the Venn diagrams.

We performed bulk miR sequencing on plasma fractions of AngII, HFD and MHS mice to assess their effects on the plasma miRNome. We did not observe significant sex-specific differences in control or MHS mice plasma miRNomes (FDR ≤ 0.05, |Log2 FC| ≥ 1 and a normalised count exceeding 10). Only one microRNA was differentially expressed between male and female control mice (miR-202-3p; enriched in females), and none were in MHS mice. The results presented afterwards thus combined data from both male and female mice. As illustrated in Figure 2A in Venn diagrams, dysregulated miRs compared to control animals of AngII, HFD or MHS mice show that many of these are common even for stresses as different as AngII and HFD. Similarities were also present for the profile of upregulated and downregulated miRs (Figure 2B–D) between AngII mice and HFD mice.

A set of 31 miRs was dysregulated by AngII, HFD, or MHS (listed in Figure 2E); 24 were upregulated, and 7 were downregulated. Using miRNet 2.0 and the TargetScan database, we identified target genes for the 24 upregulated miRs [26,27,28,29,30].

**Figure 3:**
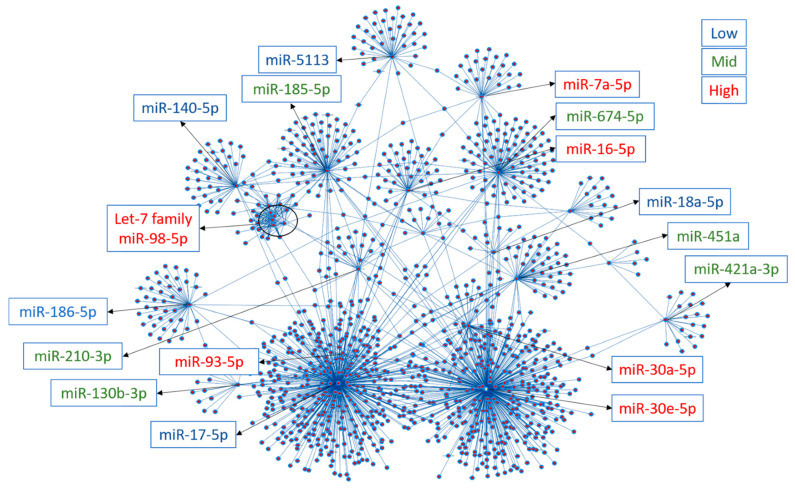
A. Interactome network of the 24 common plasma miRs with their potential target from the TargetScan database. This network was generated using the miRNet 2.0 analytical tool and Cytoscape to build the illustrated network. The location of the plasma miRs is indicated and was classed in tertiles (low, blue, mid, green, high, and red) based on their normalised counts after sequencing. Dots represent target genes.

Figure 3 illustrates a network of about 1200 possible mRNA targets for the 24 upregulated miRs. We differentiated between the highly (red), the mid (green), and the lowly (blue) expressed miRs in the network. Among the highly expressed circulatory miRs, mir-30-5p and mir-30a-5p had the most potential mRNA targets, with 382 and 51, respectively. Several signalling pathways from the possible gene targets were identified from the KEGG database. The ten KEGG pathways with the lowest false discovery rates are listed in Table S3. Interestingly, many of these gene targets were associated with the control of extracellular matrix physiology, an essential component of pathological cardiac hypertrophy, namely the transforming growth factor beta (*Tgfb1*) and its receptor (*Tgfbr1*), several collagens, *Smads*, fibroblasts growth factors (*Fgfs)*, fibronectin *(Fn1)* among others.

### 3.3. MHS Cardiac Hypertrophy is Reversed after RR

**Figure 4:**
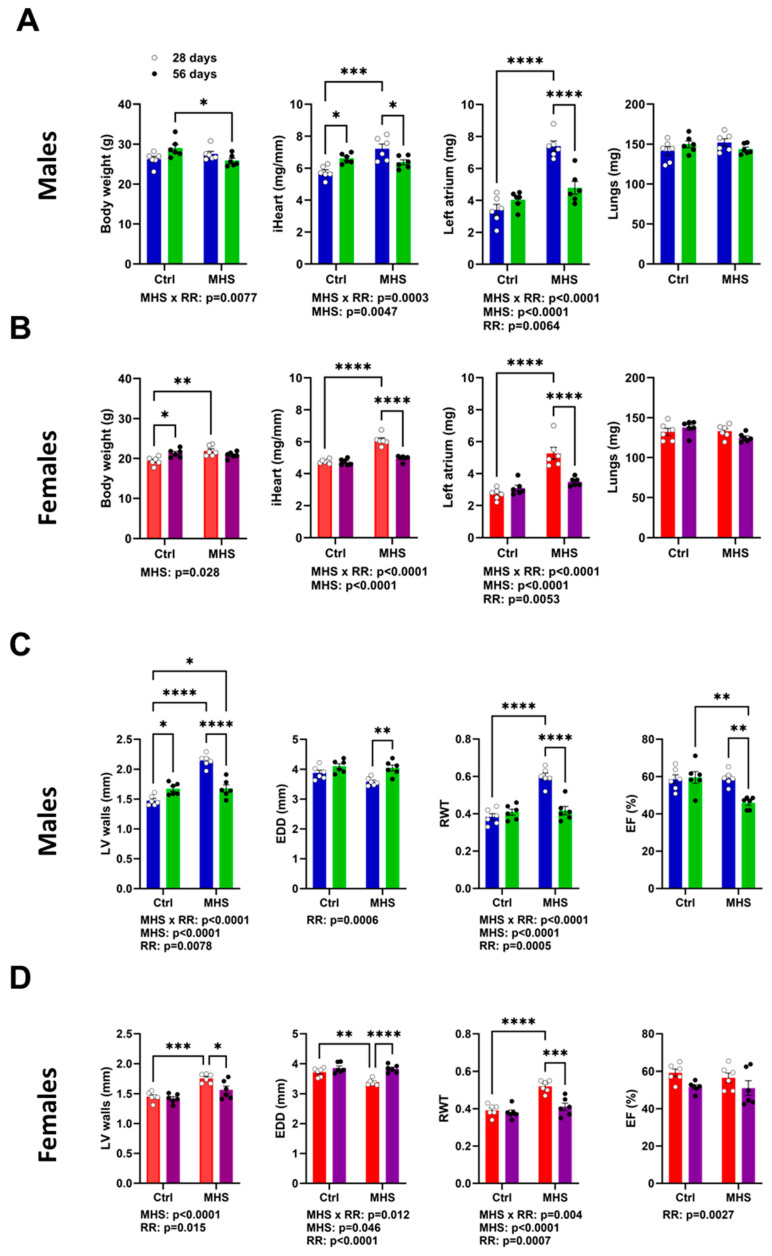
Four weeks after AngII and HFD cessation and introduction of voluntary exercise reversed cardiac hypertrophy and left atrial enlargement in male and female mice. (**A**) Males. Data at euthanasia (n = 6/group). Blue columns and open dots (28 days) and green columns and black dots (56 days). MHS increased heart and left atrial weight, and RR returned these parameters to values comparable to those of age-matched control mice. (**B**) Females. Red columns and open dots (28 days) and purple columns and black dots (56 days). MHS increased heart and left atrial weight, and RR returned these parameters to values comparable to those of age-matched control mice. (**C**) Males. Echo data. RR returned MHS-increased LV wall thickness to normal. This was true for the end-diastolic LV diameter (EDD) and relative wall thickness (RWT) but reduced ejection fraction (EF). (**D**) Females. RR returned MHS-increased LV wall thickness to normal. This was true for the end-diastolic LV diameter (EDD), and relative wall thickness (RWT) and ejection fraction (EF) were preserved. Data are represented as mean + SEM. Two-way ANOVA followed by Holm–Sidak post-test. * *p* < 0.05, ** *p* < 0.01, *** *p* < 0.001 and **** *p* < 0.0001 between indicated groups. Factors (MHS, RR of their interaction) below *p* < 0.05 are indicated below graphs.

As described above, half the MHS mice had their osmotic mini pump removed after 28 days. They were then fed a low-fat diet and allowed to VE for 28 days. CH was present after MHS was normalised after RR 28 days later (Figure 4A,B). Left atrial LA weight, which increased in MHS groups, was reduced after RR. As illustrated in Figure S1, RR improved the exercise capacity of all mice, including MHS mice, but did not normalise it.

Echo data confirmed this LV reverse remodelling, as illustrated in Figure 4C,D. LV wall thickness (IVS + PW) increased after MHS and returned to control values after RR. End-diastolic diameter was like age-matched controls, and relative LV wall thickness (RWT) also returned to control values. However, the ejection fraction in RR males was below that of controls.

### 3.4. Plasma miRNome, But Not LV miRNome, Is Normalised after RR

**Figure 5:**
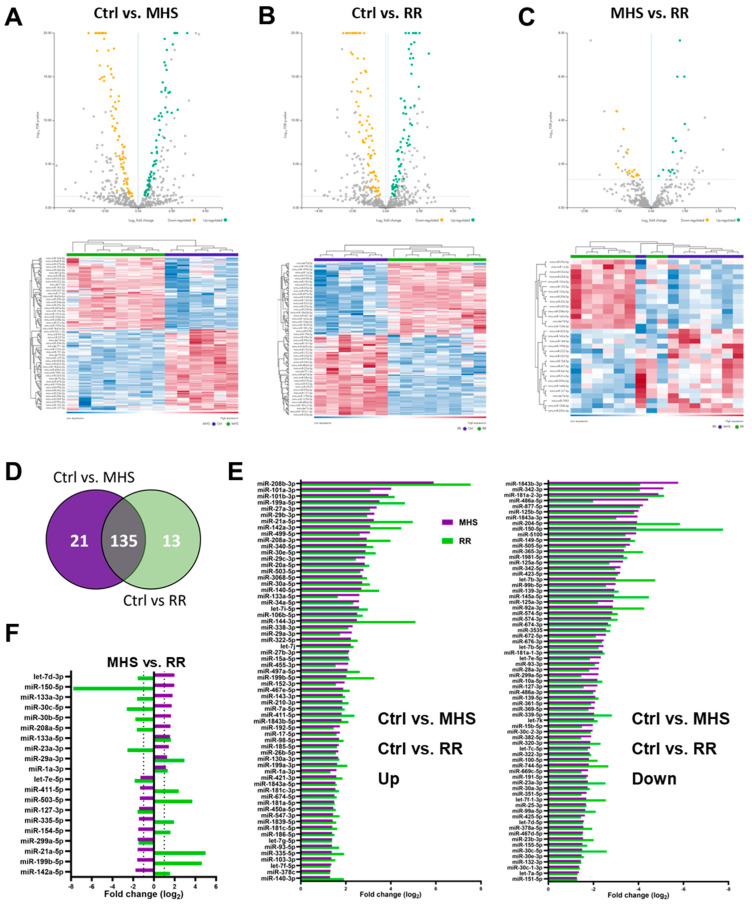
Alteration of the LV miRNome after four weeks of MHS and then RR. Volcano plots and heat map of differently expressed miRs after MHS compared to controls (**A**), RR compared to controls (**B**,**C**), between MHS and RR. Green dots illustrate upregulated miRs and yellow dots downregulated ones. Grey dots either had a fold change below ±2 or had a false discovery rate (FDR) over 0.5. Dark blue samples are controls (MHS in (**C**)), and green ones are MHS (**A**) or RR (**B**,**C**). Colours range from blue (low expression) to red (high expression). (**D**) Venn diagram illustrates the number of modulated miRs between the control group and MHS and between the control and RR groups. (MHS: purple and RR: green). (**E**) All miRs upregulated (left) and downregulated (right) between control and MHS (purple) or RR (green) in the LV are modulated in the same fashion. (**F**) Only 20 miRs are differentially modulated when comparing the LV miRNomes of the MHS group (purple) at 28 days and the RR group (green) at 56 days, and 12 of them are regulated in different directions.

We then characterised the myocardial miRNome of MHS mice and compared it to controls. We found 156 differentially expressed miRs between MHS mice and control; 83 were downregulated, and 73 were upregulated (Figure 5A). RR did not bring normalisation of the myocardial miRNome, where 148 miRs were still dysregulated compared to controls (Figure 5B). Only 31 miRs significantly differed when the MHS and the RR group were compared directly (Figure 5C). One hundred thirty-five dysregulated miRs were shared between MHS and RR mice when both groups were individually compared to controls (Figure 5D). A small portion of these miRs were less dysregulated in RR animals, and none had their expression normalised (Figure 5E).

We then identified common miRs modulated in MHS and RR mice but in the opposite direction compared to controls. Of these 20 miRs, 14 had their levels modulated toward normal levels (Figure 5F).

**Figure 6:**
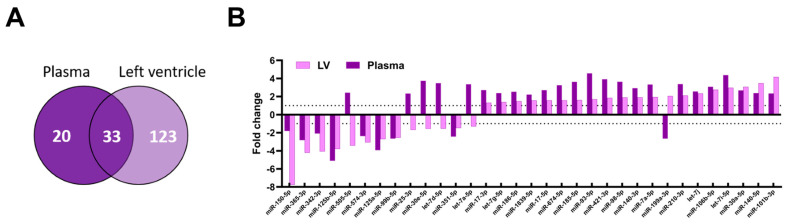
Comparison of the plasma and myocardial miRNomes. (**A**) Most plasma miRs modulated in the LV are also modulated in the plasma. In the LV, this represents only 21% of modulated miRs. (**B**). Most of the 33 common miRs between the plasma and LV miRNome of MHS-treated mice are modulated in the same direction (27 of 33).

We compared the plasma and myocardial miRNome profiles in MHS mice. Almost two-thirds (33/54) of plasma miRs dysregulated by MHS were modulated in the left ventricle, representing 13% of the myocardial dysregulated miRs (Figure 6A). Six of these thirty-three miRs were modulated differently between the plasma and the LV (Figure 6B).

**Figure 7:**
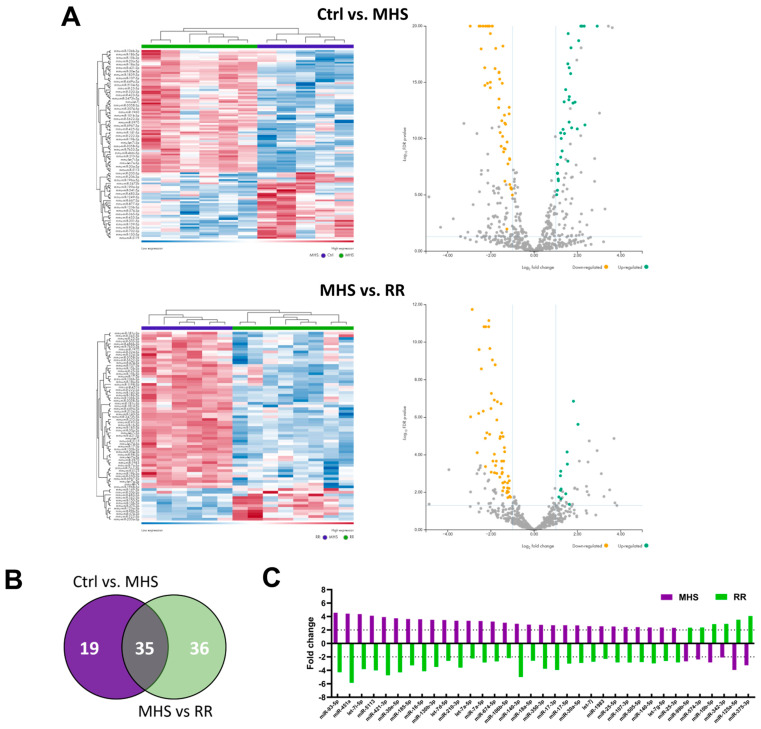
The plasma miRNome is completely normalised after RR. (**A**) Heat maps and volcano plots of differently expressed miRs after MHS (up) and RR (bottom). Green dots illustrate upregulated miRs, and yellow dots downregulated ones. Grey dots either had a fold change below ±2 or had a false discovery rate (FDR) over 0.5. (**B**) Venn diagram illustrating the number of modulated miRs between the control group and MHS and between MHS and RR groups. (MHS: purple and RR: green). (**C**) All miRs upregulated or downregulated between control and MHS (purple) are modulated in the opposite direction in the RR (green) group.

Unlike the LV miRNome, four weeks of RR was sufficient to completely normalise the circulatory miRNome after MHS (Figure 7A–C). No miRs were found to be differentially expressed in the plasma between controls and RR mice.

**Figure 8:**
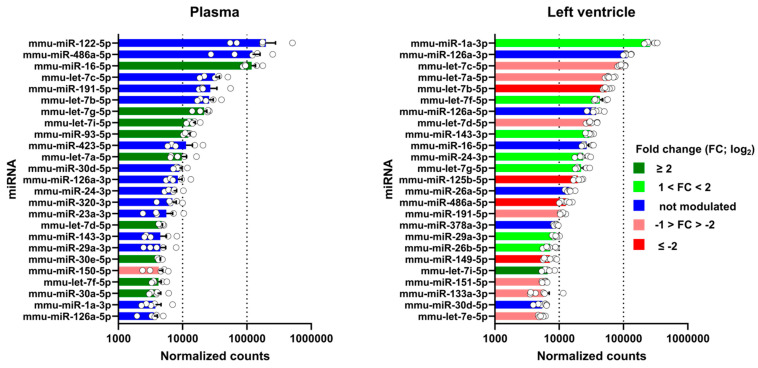
Changes in the most abundant miRs in the plasma and the LV after MHS. The list of the 25 most abundant miRs was established as the average normalised counts of the control samples from the plasma or the left ventricle. Light and dark green are the miRs upregulated after MHS, and those downregulated in pink and red. In blue, miRs where levels remained stable.

Using the normalised counts obtained from the miR sequencing, we listed the 25 most abundant miRs in the plasma and left ventricle (Figure 8). Seventeen of the twenty-five most abundant miRs in the plasma and the LV were identical. We determined their modulation, and only ten out of twenty-five were modulated in the plasma, all upregulated except for one. For the left ventricle, 19 of the most abundant miRs were modulated, 8 upregulated and 11 downregulated. Five of these were very strongly modulated (FC > ±2).

**Figure 9:**
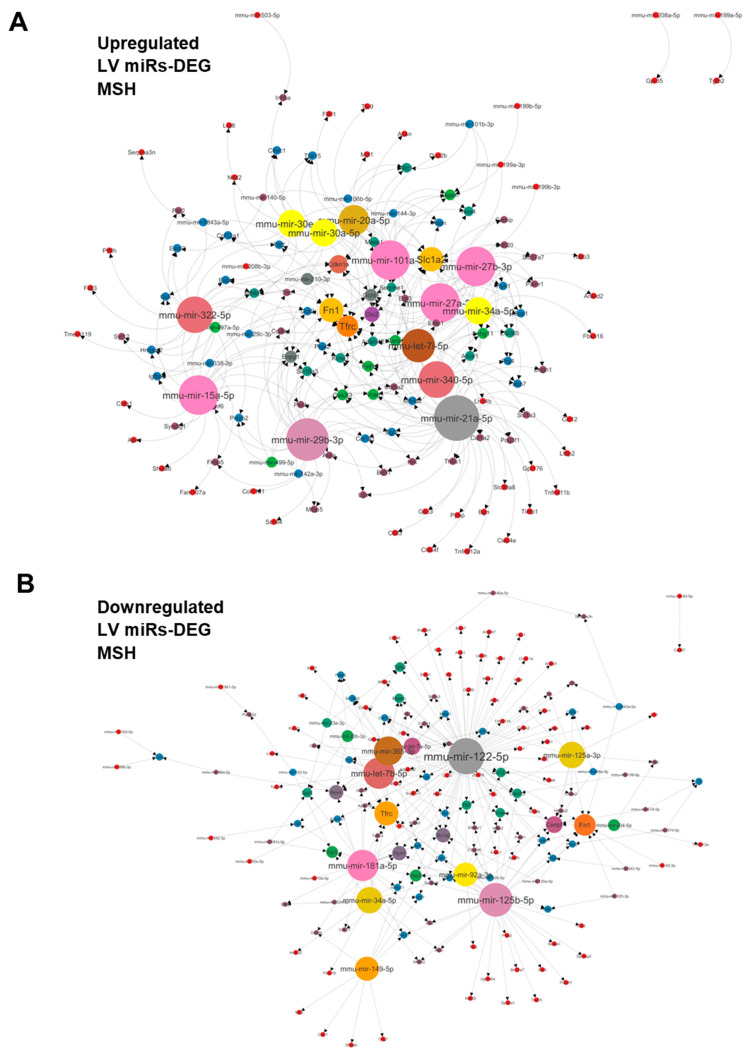
(**A**) Interactome network of 34 strongly upregulated (+log_2_2 FC) LV miRs vs. differentially regulated genes determined after a bulk RNA sequencing of the same LV RNA samples used for determining the miRNome of MHS LV compared to controls. This network was generated using the miRNet 2.0 analytical tool and Cytoscape to build the illustration. The number of possible interactions of miRs and genes determines the sizes of each dot on the network. (**B**) Interactome network of 49 strongly downregulated (−log_2_2 FC) LV miRs vs. LV differentially regulated genes after MHS compared to controls.

We then crossed the dysregulated miR profile obtained from the LV with a myocardial transcriptome from MHS mice [23]. We produced a network of miRs targeting differentially expressed genes (DEGs) in the left ventricle of MHS mice. Figure 9A,B illustrate networks for upregulated and downregulated miRs, respectively. Among the myocardial upregulated miRs, miR-21a-50, miR-29b-3p, miR-27a-3p, miR-27b-3p, miR-15a-50 and miR-101a-5p had the most mRNA targets. Fibronectin (Fn1), transferrin receptor (Tfrc), Cyclin-Dependent Kinase Inhibitor 1A (Cdkn1a), Iodothyronine Deiodinase 2 (Dio2) and Ectonucleotide Pyrophosphatase/Phosphodiesterase 1 (Enpp1) were the genes most targeted by miRs. For the downregulated myocardial miRs, mir-122-5p and mir-125-5p had the most mRNA targets, with 84 and 32, respectively. Fn1 and Tfrc were among the potentially most targeted genes by the downregulated miRs. Centromere Protein F (Cenpf), NADPH Oxidase 4 (Nox4) and Solute Carrier Family 1 Member 2 (Slc1a2) followed in this order.

## 4. Discussion

This study used a two-hit mouse model of HFpEF combining AngII and an HFD for 28 days (MHS) [23]. This model shows many similarities with clinical HFpEF, including hypertension, obesity, preserved EF, concentric LV remodelling, left atrial hypertrophy, and reduced exercise tolerance. We observed that many of these abnormalities were reversed after stopping MHS and introducing voluntary exercise for an additional 28 days (RR). Although extensive RR was observed, myocardial recovery was still incomplete. For instance, though significantly increased from voluntary exercise in all animals, exercise tolerance stayed inferior in the RR group compared to controls. Moreover, we observed that the myocardial miRNome of RR animals did not differ much from that of MHS animals four weeks before.

Our mouse model of heart failure has some strengths and weaknesses. Unlike humans, the mice in this study were young, and their heart growth was incomplete [24]. In mice, the heart continues to gain mass throughout their lives [31]. Female mice were not ovariectomised to simulate menopause, allowing for potential protection from ovarian hormones.

Angiotensin II infusion counterbalanced high-fat diet-induced obesity. Angiotensin II has been shown to induce lipolysis in mice, which opposes the HFD effects [32]. Therefore, it will be important in a future study to better characterise the changes that MHS causes to specific fat depots in mice since these produce a significant proportion of circulating miRs [33].

To study RR and potentially myocardial recovery, we used a model where HFpEF was still mild, although significant. Pulmonary congestion, a marker of heart failure, was absent except for HFD females and AngII males (*p* = 0.051). That said, from a clinical perspective, pulmonary congestion is treated with diuretics, and an intervention involving an aerobic exercise program would not be initiated in a patient with this condition untreated.

Ageing remains an unmet criterion in this study. We still do not know if reverse remodelling is as effective in an older animal with age-related myocardial abnormalities. The answer is probably no, but that does not mean some degree of reverse remodelling could not be achieved. In HFpEF patients, the benefits of exercise are associated with a decrease in hospitalisations and an increase in quality of life [34,35,36]. Cardiac benefits are less evident, suggesting that the effects we observed in our young adult mice may be less in older animals. Another difference between humans and mice is that a sedentary lifestyle is not a choice for the latter. What we call voluntary training is more of a return to a normal lifestyle for these animals [37,38,39]. This also lowers the stress of life in captivity.

Valve replacement in patients with heart valve diseases such as aortic stenosis is one of the clinical situations where regression of heart hypertrophy and structural remodelling can be observed [40]. The phenotype of aortic stenosis patients shares many features with HFpEF patients. In these patients, depending on the level of heart damage present before the procedure, only a tiny proportion will see their condition improve in one year, and for the most part, the damage will remain stable [41]. This is the case even if almost all patients show some hypertrophy regression levels [40]. The reasons why some individuals see their clinical state improve or remain stable and others deteriorate with similar heart damage to begin with remain unclear [41].

We observed that myocardial and plasma miRNome did not respond similarly to the four weeks granted for myocardial recovery. The circulatory miRNome seemed to return to normal, but not the LV one, suggesting that abnormal processes were still active in the myocardium. Two hypotheses can be offered here. The first is that the changes in the plasma and myocardial miRNomes observed following MHS were part of a reaction seeking to reduce the harmful effects of the stress and that despite the four weeks of recovery, myocardial repair or protection processes were still necessary. The second hypothesis is that the remaining alterations in the LV miRNome point toward abnormalities that are now irreversible. Both hypotheses are probably true in proportions that are still difficult to establish.

The data obtained from transcriptomic studies or, here, on microRNAs rarely succeed in pointing to a single cause for a phenotype. Our observations highlight the complexity of the phenomena and mechanisms involved and their interrelations.

Nevertheless, some observations seem essential to emphasise. Despite a myocardial miRNome profile still essentially abnormal, the circulating one now seemed to have returned to normal, suggesting that the contribution of cardiac miRs to the plasma miRNome is probably low. In addition, non-cardiac tissues appear after RR to release a normalised microRNA content into the circulation. This does not mean that other factors (proteins, other coding or non-coding RNAs, peptides…) produced and released by these tissues have also returned to normal, and this will have to be investigated in the future.

When we separate the MHS into its entities, namely AngII, a vasoconstrictor and hypertensive factor with direct pro-hypertrophic effects on the heart but also at the kidney level and the HFD, an obesogenic stress, probably inducing some insulin resistance and alterations on adipose and liver tissues, we find ourselves in front of two highly similar plasma miRNomes. This does not mean that the tissues contributing significantly to the plasma miRNome respond similarly to these stresses but that the resultant in circulation is mostly identical. This is surprising, considering the adipose tissue is a primary producer of circulating microRNAs. Stresses with opposite effects on the visceral fat, such as AngII and HFD, did not result in miRNome profiles with marked differences.

We identified more than 215 miRs out of over 1900 measured, modulated by the various stresses, and possibly involved in the following cardiac remodelling and myocardial recovery. Each of these miRs has several possible gene targets, making the total number very high. On the other hand, not all plasma miRs directly influence cardiac physiology and not all target genes are expressed significantly in the myocardium. In addition, other molecules, such as long non-coding RNAs acting as sponges and counterbalancing miRs effects, have not been studied here but probably would help offer a more complete picture [42].

Some miRs or families of miRs stand out as modulated in several of our experimental groups. Although many of them have been described before as associated with cardiac physiology, a more direct demonstration of their targets in the heart remains to be shown.

High levels of miR in the post-MHS myocardium may reflect both their inhibitory action on the expression of a factor associated with normal physiology or a way to inhibit a harmful stress-induced factor. One such example where levels of a miR can lead to such conclusions is let-7i. Both in the myocardium and in the circulation, let-7i levels are increased after MHS. Myocardial levels remained high after the recovery period. Yet, let-7i has been shown to have lower levels in the heart of mice receiving AngII for 3 or 7 days, and its administration can reduce the expression of several genes associated with fibrosis and protect the heart [43]. It can be speculated that over a more extended period of exposure to MHS, the myocardium induces the expression of protective miRs to attenuate the pro-fibrotic action of chronically administered AngII. If let-7i has an anti-fibrotic action in the heart, as mentioned above, but also in the lungs or arteries, it is instead a pro-fibrotic action that seems to have let-7i in the kidneys [44]. These observations suggest that processes involving the conventional TGFβ signalling pathway would be modulated inversely by the same miR.

Moreover, in the let-7 family, five (let-7a to let-7e) saw their levels decrease by the MHS and the reverse was observed for let-7f, g and i. This family of miRs has a highly conserved sequence and shares many possible targets [45]. Therefore, it seems that the different members of this family may differ in their actions or intervene at various times during stress, as possibly here for MHS.

Focusing on a miR or a family of these is probably reductive to understanding the stress-related action leading to heart failure, as in our mice. However, these miRs remain potential plasma biomarkers to follow the evolution of a condition or treatment over time. Unfortunately, the answers obtained from circulating miR changes can be falsely reassuring. This study shows significant molecular myocardial abnormalities remain despite seemingly complete reverse remodelling at the morphological and functional levels and a normalised plasma miRNome.

We intended to identify differentially modulated miRs between male and female mice after MHS and RR. In the end, there were few sex differences, and we studied all the animals together. However, the heart response to the high-fat diet differed between male and female mice, the latter being more sensitive to this stress.

### 4.1. Future Perspectives

This study highlights an aspect of interest: inter-organ communication during the development of HFpEF and possibly its treatment. Plasma microRNAs are only a part of the messages sent and received in these exchanges. Two recent publications have shown that inactivation of visceral white adipose tissue in mice or activation of brown adipose tissue can reverse the pro-hypertrophic action of AngII on the heart. In the case of epididymal white adipose tissue, its removal, blocking the formation of exosomes or administering miR-23a-3p, could stop the pro-hypertrophic action of AngII. In our study, miR-23a-3p was not modulated by MHS but was downregulated by AngII after 28 days [46].

For the brown adipose tissue, blocking its action through β3-adrenergic receptor knock-out increased cardiac myocardial hypertrophy and fibrosis induced by AngII, while activating an agonist had a protective effect. The adverse impact of this gene knock-out was mediated by the inducible nitric oxide synthase (iNOS) in exosomes produced by brown adipose tissue [47].

### 4.2. Study Limitations

This study used a two-hit model to induce HFpEF that resembles human HFpEF [23]. Although we were able to design and develop a murine model that includes hypertension and metabolic stress and studied it in both sexes, the model is still not representative of the entire spectrum of HFpEF patients. The number of comorbidities in patients is usually more significant. HFpEF patients are typically old, postmenopausal (women), and often suffer from atrial fibrillation, risk factors that we did not or could not include [2,3].

We did not monitor our mice’s running activity. The animals were housed in groups of 3–5; thus, it would not have been possible. Since all trained mice showed better exercise capacity than untrained ones, we assume that VE was accompanied by increased physical activity. Also, it is well-known that females run more kilometres per day than males [48], but this did not result in better resistance to an exhaustion test.

It is probable that investigating the miRNomes after four weeks of MHS or waiting four weeks after its cessation may have led us to miss the early changes in different miRs that had dissipated with time.

We chose not to concentrate on one miR over another by treating mice with exogenous miRs or blocking its action. We were interested in changes in the overall profile with the evolution of HFpEF or reverse remodelling. By doing so, we did not focus on a possible mechanism. Our study emphasises the need to keep this multi-organ view of HFpEF, a multifactorial syndrome.

## 5. Conclusions

In conclusion, we showed extensive LV reverse remodelling after MHS was stopped and voluntary exercise was introduced in a cardiometabolic HFpEF murine model. This microRNA sequencing study shows that RR is associated with normalising the plasma miRNome but not the myocardial one.

## Data Availability

The raw data supporting the conclusions of this article will be made available by the authors on request.

## Supplementary Materials

The following supporting information can be downloaded at: https://www.mdpi.com/article/10.3390/biom14080892/s1, Table S1: Echo data after AngII or HFD in in male and female mice. Table S2: Reverse remodeling after MHS. Table S3: Interactome network of the 24 common plasma miRs with their potential targets from the TargetScan database. Figure S1: Stopping MHS after 28 days and introducing voluntary exercise for an additional 28 days (total: 56 days) increases the total distance run by mice in an exhaustion protocol.
