# Supplementary material for: Plasma and Myocardial miRNomes Similarities and Differences during Cardiac Remodelling and Reverse Remodelling in a Murine Model of Heart Failure with Preserved Ejection Fraction
Source: Biomolecules. 2024 Jul 24;14(8):892. doi: 10.3390/biom14080892 (PMC11351983; doi:10.3390/biom14080892)
Supplement: Supplementary file 1 [file biomolecules-14-00892-s001.zip › biomolecules-3099403-supplementary.pdf]

Plasma and myocardial miRNomes similarities and differences during remodelling and reverse remodelling in a two-hit murine model of heart failure with preserved ejection fraction.

Sara-Ève Thibodeau[1], Emylie-Ann Labbé[1], Élisabeth Walsh-Wilkinson, Audrey Morin-Grandmont, Marie Arsenault, Jacques Couet*

Groupe de recherche sur les valvulopathies, Centre de recherche de l'Institut universitaire de cardiologie et de pneumologie de Québec, Université Laval, Québec City, Québec, Canada

Supplemental data

Table S1. Echo data after AngII or HFD in in male and female mice. Standard echo left ventricle parameters measured in mice four weeks of AngII infusion or high-fat diet (HFD) (Day 28). Control mice were also studied. Echo exams were performed on the same animals described in the Methods section. PW: diastolic posterior wall thickness, IVS: inter-ventricular septum. EDV: end-diastolic volume, ESV: end-systolic volume, SV stroke volume, HR: heart rate, CO: cardiac output. Results are expressed as the mean ± standard error of the mean (SEM) (n=6). One-way ANOVA analysis and Holm-Sidak post-test. a: p<0.05, b: p<0.01, c: p<0.001 and d: p<0.0001 vs. controls.

	**Controls**		**AngII**		**HFD**	
Parameters	Males	Females	Males	Females	Males	Females
PW, mm	0.75±0.014	0.71±0.024	1.02±0.028[d]	0.87±0.02 [c]	0.87±0.025[b]	0.88±0.022[c]
IVS; mm	0.75±0.013	0.70±0.020	0.92±0.028[d]	0.83±0.030[c]	0.87±0.025[c]	0.78±0.021[a]
HR, bpm	498±10.4	515±11.2	527±8.43	521±13.7	461±30.3	502±14.9
EDV, µl	54±3.1	47±3.0	53±3.4	44±3.5	52±2.3	54±3.8
ESV, µl	23±0.9	17±1.5	19±1.9	20±2.5	20±2.3	23±2.2[a]
SV, mm	31±2.2	30±1.7	34±2.1	25±1.7	32±2.9	31±2.8
CO mm/min	15.3±1.1	15.4±0.9	17.8±1.4	13.0±1.23	15.1±0.9	15.1±1.1

Table S2. Reverse remodeling after MHS. Standard echo left ventricle parameters were measured in male and female mice at two-time points: four weeks of MHS and 4 weeks after MHS cessation and introduction of voluntary exercise (RR) animals. Echo exams were performed on the same animals as described in the Methods section. PW: diastolic posterior wall thickness, IVS: inter-ventricular septum. EDV: end-diastolic volume, ESV: end-systolic volume, SV stroke volume, HR: heart rate, CO: cardiac output. Results are expressed as the mean ± standard error of the mean (SEM) (n=6). Two-way ANOVA analysis and Holm-Sidak post-test using data in young as controls. a: $p<0.05$, b: $p<0.01$, c: $p<0.001$ and d: $p<0.0001$ vs. control group for each time (28 or 56 days).

**Males**				
Parameters	Ctrl 28	MHS	Ctrl 56	RR
PW, mm	0.80±0.012	1.14±0.039^{d}	0.84±0.023	0.87±0.044
IVS, mm	0.77±0.031	0.99±0.058^{d}	0.82±0.026	0.81±0.026
EDV, µl	63±4.7	49±3.3^{c}	64±3.7	62±2.5
ESV, µl	29±3.6	22±1.8	26±3.3	33±1.5
SV, mm	34±3.5	27±1.6	38±1.5	28±1.4^{a}
HR, bpm	467±10.2	512±14.3^{a}	456±25.6	497±36.6
CO, ml/min	15.8±2.0	13.8±1.1	17.3±0.63	13.9±0.84^{b}
**Females**				
Parameters	Ctrl 28	MHS	Ctrl 56	RR
PW, mm	0.74±0.021	0.94±0.022^{d}	0.71±0.018	0.71±0.014
IVS, mm	0.72±0.011	0.94±0.066^{d}	0.71±0.016	0.64±0.012
EDV, µl	45±3.5	44±4.8	56±1.8^{a}	52±2.8
ESV, µl	19±0.9	19±1.9	27±0.8^{b}	24±2.9
SV, mm	26±1.7	25±3.3	29±1.3	28±2.1
HR, bpm	482±23.5	498±17.9	464±19.9	505±17.8
CO, ml/min	12.5±1.04	12.5±1.7	13.4±0.36	14.3±1.2

Table S3. Interactome network of the 24 common plasma miRs with their potential targets from the TargetScan database. Ten most significant pathways from the KEGG database.

Kegg pathway	Hits	FDR	Target genes
Pathways in cancer	54	5.47E-09	Akt3, Apc, Apc2, Araf, Arnt2, Bcl2, Bmp4, Ccnd1, Ccne1, Ccne2, Col4a2, Crkl, Ctbp2, Ctnnb1, Cxcl12, Egfr, Egln2, Ep300, Fgf10, Fgf13, Fgf2, Fn1, Gnai2, Gnai3, Gng2, Gng4, Hif1a, Igf1, Igf1r, Il6, Itgav, Jun, Kras, Lpar3, Mdm2, Myc, Nras, Prkcb, Pten, Ralgds, Rb1, Runx1, Shh, Smad2, Smad3, Stat3, Tcf7, Tcf7l2, Tgfa, Tgfb1, Tgfbr1, Vegfa, Wnt3a. Xiap
MAPK signaling pathway	39	2.85E-08	Akt3, Atf2, Bdnf, Cacna2d1, Cacng7, Crkl, Dusp7, Egfr, Fgf10, Fgf13, Fgf2, Flnb, Il1a, Jun, Kras, Map3k1, Map3k12, Map3k5, Map4k2, Mapk14, Mef2c, Mknk1, Mknk2, Myc, Nf1, Nfatc3, Nras, Pla2g4b, Ppp3ca, Ppp3cb, Ppp3r1, Prkcb, Rasa1, Rps6ka3, Rps6ka5, Taok1, Taok2, Tgfb1, Tgfbr1
Prostate cancer	21	3.47E-08	Akt3, Araf, Bcl2, Ccnd1, Ccne1, Ccne2, Creb1, Creb5, Ctnnb1, Egfr, Ep300, Igf1, Igf1r, Kras, Mdm2, Nras, Pten, Rb1, Tcf7, Tcf7l2, Tgfa
Signaling pathways regulating pluripotency of stem cells	27	3.95E-08	Acvr1b, Akt3, Apc, Apc2, Bmp4, Bmpr2, Ctnnb1, Fgf2, Id1, Id4, Igf1, Igf1r, Inhba, Klf4, Kras, Mapk14, Myc, Nras, Skil, Smad1, Smad2, Smad3, Smad5, Stat3, Tbx3, Wnt3a, Zfhx3
FoxO signaling pathway	26	6.22E-08	Akt3, Araf, Bcl2l11, Ccnd1, Ccnd2, Egfr, Ep300, Homer2, Igf1, Igf1r, Il6, Il7r, Klf2, Kras, Mapk14, Mdm2, Nras, Prkaa1, Pten, Rbl2, Setd7, Smad2, Smad3, Stat3, Tgfb1, Tgfbr1
Colorectal cancer	17	1.11E-07	Akt3, Apc, Apc2, Araf, Bcl2, Ccnd1, Ctnnb1, Jun, Kras, Myc, Ralgds, Smad2, Smad3, Tcf7, Tcf7l2, Tgfb1, Tgfbr1
Hepatitis B	26	3.71E-07	Akt3, Atf2, Bcl2, Ccnd1, Ccne1, Ccne2, Creb1, Creb5, Ddx3x, Ddx58, Ep300, Ifnar1, Il6, Jun, Kras, Map3k1, Myc, Nfatc3, Nras, Prkcb, Pten, Rb1, Stat3, Tgfb1, Tgfbr1, Ywhaz
PI3K-Akt signaling pathway	45	6.41E-07	Akt3, Atf2, Bcl2, Bcl2l11, Ccnd1, Ccnd2, Ccne1, Ccne2, Col1a2, Col4a2, Creb1, Creb5, Csf1, Ddit4, Egfr, Eif4e, Fgf10, Fgf13, Fgf2, Fn1, Gng2, Gng4, Ifnar1, Igf1, Igf1r, Il6, Il7r, Itgav, Itgb8, Kras, Lpar3, Mcl1, Mdm2, Myc, Nras, Osmr, Phlpp1, Ppp2r2c, Prkaa1, Pten, Rbl2, Rps6kb1, Vegfa, Ywhag, Ywhaz
Proteoglycans in cancer	31	1.21E-06	Akt3, Araf, Camk2d, Cav1, Ccnd1, Col1a2, Ctnnb1, Egfr, Erbb4, Fgf2, Flnb, Fn1, Hif1a, Igf1, Igf1r, Itgav, Kras, Mapk14, Mdm2, Myc, Nras, Prkcb, Ptpn11, Rdx, Rps6kb1, Smad2, Stat3, Tgfb1, Timp3, Vegfa,Wnt3a
HIF-1 signaling pathway	21	1.33E-06	Akt3, Bcl2, Camk2d, Egfr, Egln2, Eif4e, Eno2, Ep300, Hif1a, Ifngr1, Igf1, Igf1r, Il6, Mknk1, Mknk2, Prkcb, Rps6kb1, Serpine1, Stat3, Timp1, Vegfa

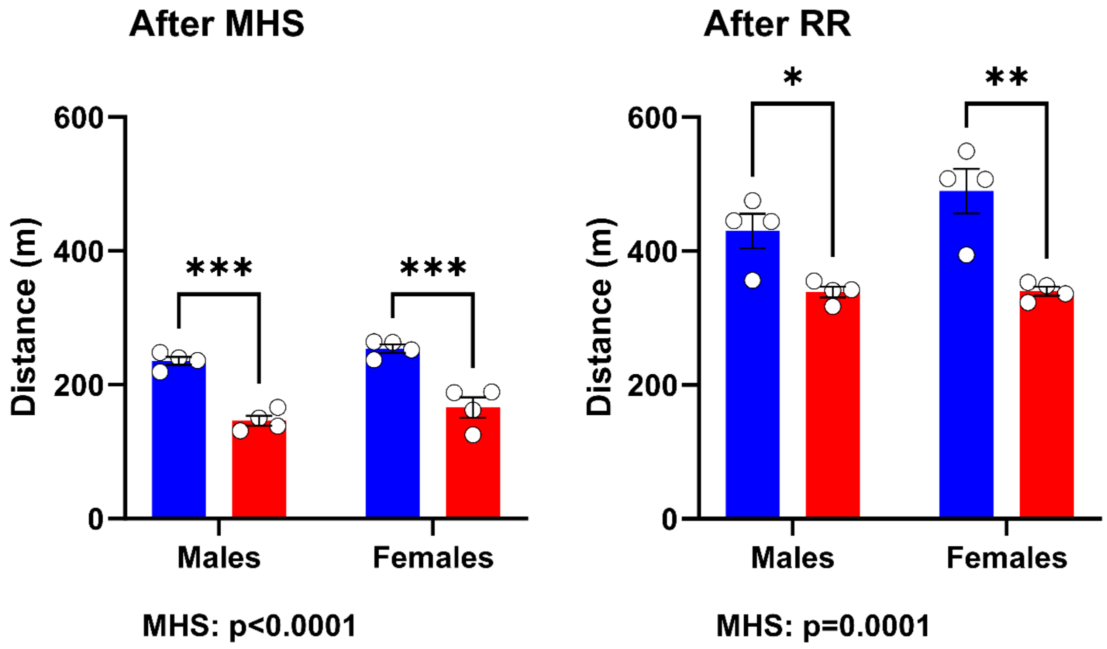

Figure S1. Stopping MHS after 28 days and introducing voluntary exercise for an additional 28 days (total: 56 days) increases the total distance run by male (blue) and female (red) mice on a treadmill in an exhaustion protocol. Two-way ANOVA was followed by the Holm-Sidak post-test (n = 4/groups). Significant variables after two-way ANOVA are indicated below the graphs. *: p<0.05, **: p<0.01 and ***: p<0.001 between the indicated groups by brackets.
